# Synergistic Effect of Saccharin and Caffeine on Antiproliferative Activity in Human Ovarian Carcinoma Ovcar-3 Cells

**DOI:** 10.3390/ijms241914445

**Published:** 2023-09-22

**Authors:** Sun Ju Lee, Sang-Yong Park, Subin Bak, Min-Woo Lee, Dae Jin Lim, Hyeong-Dong Kim, Dong-Gil Kim, Suhng Wook Kim

**Affiliations:** 1 Department of Health and Safety Convergence Science, Graduate School, Korea University, 145 Anam-ro, Seoul 02841, Republic of Koreablast.lim@samsung.com (D.J.L.);; 2 BK21 FOUR R&E Center for Learning Health Systems, Korea University, 145 Anam-ro, Seoul 02841, Republic of Korea; 3 Department of Laboratory Medicine and Genetics, Samsung Medical Center, Seoul 06351, Republic of Korea; 4 Kyung-In Synthetic Corporation, 572 Gonghang-daero, Seoul 07947, Republic of Korea; 5 Graduate School of Particulate Matter Specialization, Korea University, 145 Anam-ro, Seoul 02841, Republic of Korea

**Keywords:** Ovcar-3 cell, saccharin, caffeine, synergistic effect

## Abstract

The purpose of this study was to confirm the antiproliferative and apoptotic induction potential of a saccharin and caffeine combination in ovarian cancer cells. The cell line used was Ovcar-3, and the cell viability was measured through a WST-8 assay, while a Chou–Talalay assay was used to confirm the synergistic effect of saccharin and caffeine on the ovarian cancer cells. A clonogenic assay, annexin V-FITC/PI-PE double-staining, and RT-PCR were performed to confirm the expression of genes that induce colony formation, cell viability, and apoptosis in ovarian cancer cells treated with the saccharin–caffeine combination. It was demonstrated that both saccharin and caffeine decreased the viability of Ovcar-3 cells, and the cell viability decreased even more significantly when the cells were treated with the combination of saccharin and caffeine. The clonogenic assay results showed that the number of colonies decreased the most when saccharin and caffeine were combined, and the number of colonies also significantly decreased compared to the single-treatment groups. Based on flow cytometry analysis using annexin V-FITC/PI-PE double-staining, it was confirmed that the decrease in cell viability caused by the combination of saccharin and caffeine was correlated with the induction of apoptosis. The results of the RT-PCR confirmed that the combined treatment of saccharin and caffeine promoted cell apoptosis by regulating the expression of apoptosis-inducing genes. These results demonstrate that the combination of saccharin and caffeine more efficiently inhibits the proliferation of Ovcar-3 cells and induces apoptosis in vitro.

## 1. Introduction

Epithelial ovarian cancer is among the five cancers with the highest mortality rate in the United States [1]. As ovarian cancer has no clear symptoms in its early stages, many patients are diagnosed with terminal cancer [2]. Hence, the mortality rate is high, as the symptoms appear only after it has already spread widely within the peritoneum [3]. Ovarian cancer is histologically differentiated into different types according to the lesions it develops [3]. Among them, epithelial cell ovarian cancer accounts for over 90% of the incidence of ovarian cancer [4], and Ovcar-3 cells are widely used to study epithelial-cell-derived ovarian cancer. In recent years, the majority of patients with high-grade serous ovarian cancer have been resistant to chemotherapy, and this phenomenon has accounted for the majority of deaths from epithelial ovarian cancer [5]. Hence, the development of new strategies for chemotherapy resistance has emerged as an important research task [6].

An artificial sweetener, saccharin, is about 300 times sweeter than sugar and, as a calorie-free sugar surrogate, it is being established as a sugar substitute in diabetic patients [7]. While a study in 1977 demonstrated that male rats fed saccharin had an increased risk of bladder cancer, in 1993, the World Health Organization declared it a safe sweetener for humans, and the International Agency for Research on Cancer and the American Toxicology Program excluded it from the list of carcinogens. The US Food and Drug Administration lifted the ban in 2001. Saccharin is primarily used in the form of a sodium salt soluble in water [8], and as an alternative raw material for sugar. It is also used in soft drinks, diet foods, sweets, and many commercial products, including lip balm and toothpicks [7]. While the safety of saccharin was controversial in the 1970s, the risk to human health of saccharin intake at concentrations in the normal range was not clearly established [8]. Saccharin is part of the sulfonamide family, and it has already been reported that this sulfonamide-based substance acts as an antitumor substance. Hence, its potential for use as a treatment for solid cancer has been reported [9]. 

Caffeine, part of the methyl xanthine family, is found in coffee, tea, cocoa, cola, etc., and is among the most widely consumed psychoactive substances in the world [10,11]. On average, 150 mL of coffee contains about 120 mg of caffeine, while black tea and soft drinks contain about 50 mg and 30–60 mg, respectively [11]. Studies have reported various pharmacological effects of caffeine [11]. Caffeine interferes with the binding of adenosine-to-adenosine receptors and binds to adenosine receptors, thereby activating the response of norepinephrine, dopamine, and serotonin, further disrupting sleep [11]. Furthermore, caffeine interferes with the normal growth of cancer cells by inhibiting the activity of ataxia-telangiectasia-mutated and ataxia-telangiectasia-related kinases that arrest the cell cycle or induce apoptosis when the DNA in cells is damaged [12,13]. As such, various studies suggest caffeine as an anticancer agent for some types of cancer [13,14,15,16]. 

However, no study on whether the combination of saccharin and caffeine has a synergistic effect on Ovcar-3 cells has yet been reported. Hence, in this study, saccharin and caffeine were combined to confirm the possibility of inducing antiproliferative activity and apoptosis in ovarian cancer cells.

## 2. Results

### 2.1. Anti-Proliferative Activity of Saccharin or Caffeine Single-Drug Treatment in Ovcar-3 Cells

**Figure 1:**
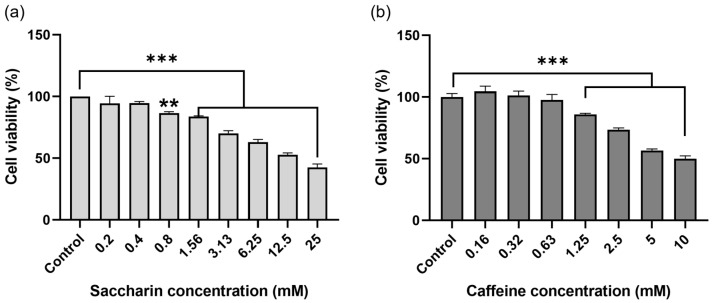
Cell viability of Ovcar-3 in response to treatment with saccharin (SC) and caffeine (CA). Graphs showing relative survival following 24 h incubations with (**a**) SC (0, 0.2, 0.4, 0.8, 1.56, 3.13, 6.25, 12.5, 25 mM) and (**b**) caffeine (0, 0.16, 0.32, 0.63, 1.25, 2.5, 5, 10 mM) in Ovcar-3. Each experiment was performed in triplicate; *** *p* < 0.001, ** *p* < 0.01.

Ovcar-3 cells were treated with saccharin or caffeine for 24 h, and the cell viability was confirmed via a WST-8 assay. When cells were treated with saccharin by concentration, the survival rate of Ovcar-3 cells decreased in a concentration-dependent manner compared to the cells not treated with saccharin, and a statistically significant decrease (*p* < 0.001) was confirmed (Figure 1a). Furthermore, among the Ovcar-3 cells treated with caffeine, the cell viability decreased in a concentration-dependent manner compared to the cells not treated with caffeine, and a statistically significant decrease (*p* < 0.001) was confirmed (Figure 1b).

### 2.2. Anti-Proliferative Activity of Saccharin and Caffeine Combination Treatment in Ovcar-3 Cells

**Figure 2:**
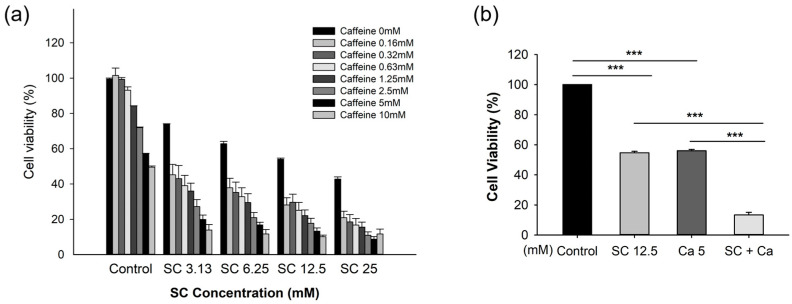
The effect of saccharin (SC) with caffeine (CA) on cell viability among Ovcar-3 cells. (**a**) The combination effects of concentrations of saccharin (3.13, 6.25, 12.5, 25 mM) and caffeine (2.5, 5, 10 mM). (**b**) The combination effects of saccharin (12.5 mM) with caffeine (5 mM) on cell viability among Ovcar-3 cells. Each experiment was performed in triplicate; *** *p* < 0.001.

To confirm the synergistic effect of the saccharin–caffeine combination, the cell viability was confirmed via treating the Ovcar-3 cells with various concentrations of saccharin (3.13, 6.25, 12.5, 25 mM) and caffeine (2.5, 5, 10 mM) to assess cell viability (Figure 2a). As demonstrated in Figure 2b, the cell viability in the saccharin (12.5 mM) single-treatment group was 52.6 ± 0.027%, and a statistically significant decrease in cell viability was demonstrated compared to the cells not treated with saccharin (*p* < 0.001). Furthermore, the cell viability in the caffeine (5 mM) single-treatment group was 56.7 ± 1.175% (*p* < 0.001), and a statistically significant decrease in cell viability was demonstrated compared to the cells not treated with caffeine (*p* < 0.001). Furthermore, in the group treated with saccharin (12.5 mM) and caffeine (5 mM), the cell viability was 11.9 ± 4.215%, which was significantly decreased compared to the groups treated with either saccharin or caffeine alone, and a statistically significant decrease was demonstrated (*p* < 0.001). 

**Figure 3:**
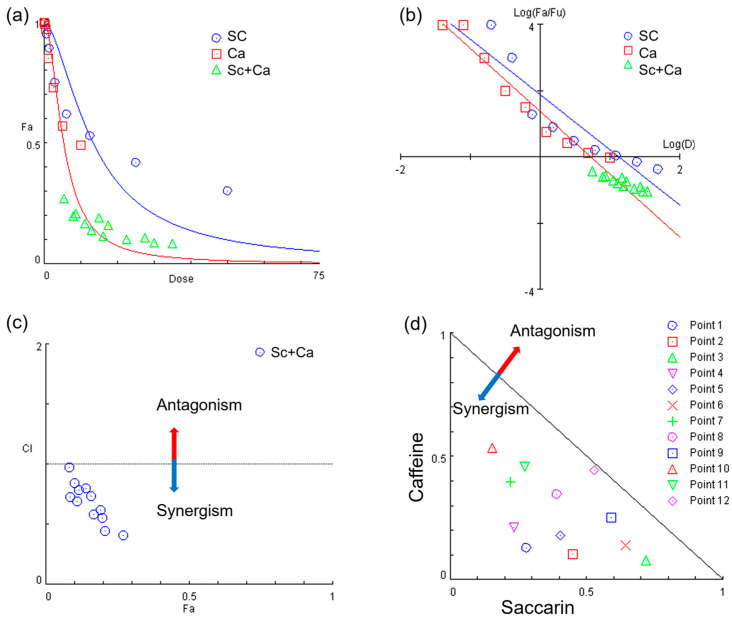
(**a**) Dose–effect curve, (**b**) median-effect plot, (**c**) Fa-combination-index plot, (**d**) combination index (CI) values calculated using CompuSyn (version 1.0); a CI value < 1 indicates a synergistic effect between drugs, and a CI value > 1 indicates an antagonistic effect between drugs. The cells were analyzed using CompuSyn version 1.0 (ComboSyn, Inc., Paramus, NJ, USA).

The utilization of a constant ratio combination facilitates the computerized simulation of dose–response curves (Figure 3a), median-effect plots (Figure 3b), Fa-combination-index plots (Figure 3c), and isobolograms (Figure 3d). Figure 3a illustrates the concentration-dependent cytotoxicity analysis of either single-treatment saccharin and caffeine or their combination against Ovcar-3 cells. As shown in Figure 3d, as all of the 12 combination data points are on the synergy side (CI < 1) and show substantial synergism. As all CI values were <1, it was determined that the decrease in cell viability due to the combination of saccharin and caffeine was caused by the synergistic effect.

### 2.3. Clonogenic Assay

**Figure 4:**
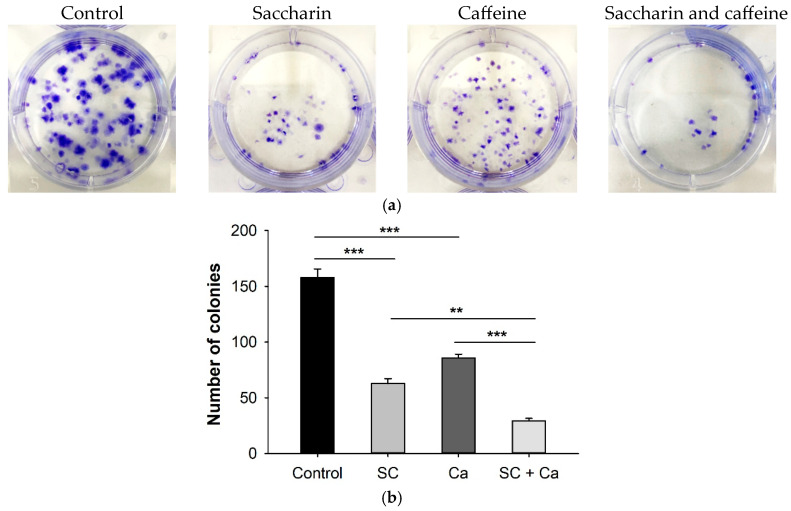
(**a**) Ovcar-3 cells were treated with saccharin (SC) (12.5 Mm) and caffeine (CA) (5 Mm) alone or in combination. The colonies were stained with crystal violet. Representative images of the clonogenic assays are shown. (**b**) Graph showing the number of colonies. Data are presented as mean ± standard error. Each experiment was performed in triplicate; *** *p* < 0.001, ** *p* < 0.01.

Colony formation was observed, to determine whether the combination of saccharin and caffeine affected the colony formation of Ovcar-3 cells (Figure 4a). In both the saccharin and caffeine single-treatment groups, the number of colonies decreased compared to the control, and a statistically significant decrease was observed (Figure 4b). In particular, the combination of saccharin and caffeine demonstrated the largest decrease in the number of colonies, and the number of colonies was significantly decreased even compared to the groups treated with either saccharin or caffeine alone.

### 2.4. Apoptosis Induced by Saccharin Combined with Caffeine in Ovcar-3 Cells

**Figure 5:**
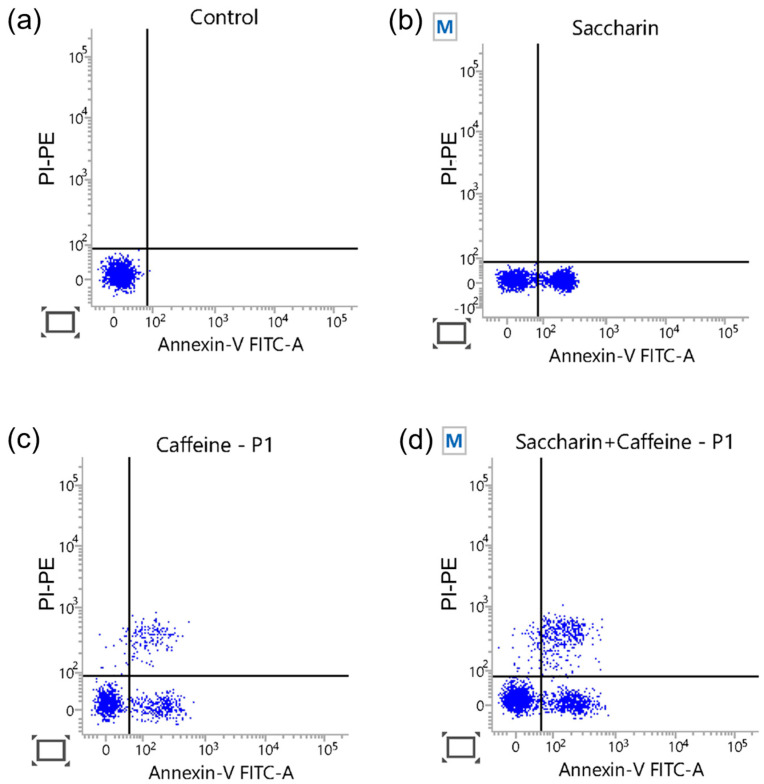
The flow cytometric detection of apoptosis with annexin V-FITC/PI-PE double-staining for cells: (**a**) the control, (**b**) saccharin (12.5 mM), (**c**) caffeine (5 mM), and (**d**) the combination, for 24 h in Ovcar-3 cells.

To confirm that the decrease in cell viability due to the saccharin–caffeine combination in the Ovcar-3 cells was a result of apoptosis, flow cytometry analysis using annexin V-FITC/PI-PE double-staining was performed. As demonstrated in Figure 5, the combination of saccharin and caffeine induced a higher level of apoptosis than either treatment alone. Hence, it is apparent that the decrease in cell viability due to the combination treatment of saccharin and caffeine was related to the induction of apoptosis.

### 2.5. Reverse Transcription Polymerase Chain Reaction (RT-PCR) 

**Figure 6:**
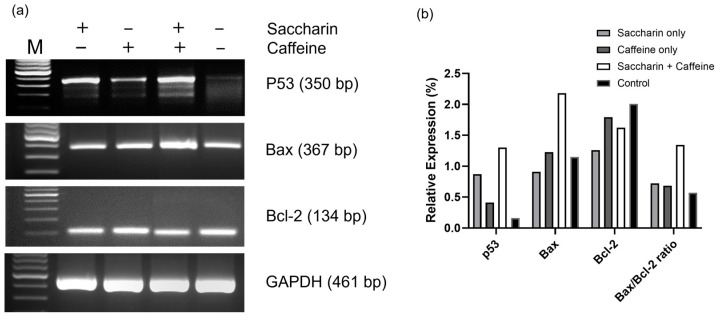
(**a**) The results of the RT-PCR analysis following treatment with saccharin and caffeine alone or in combination. (**b**) The relative mRNA expression levels of p53, BAX, Bcl-2 genes, and the BAX/Bcl-2 ratio. The RT-PCR products obtained from all groups were separated via 2% agarose gel electrophoresis with 100 ng/mL of ethidium bromide.

The expression levels of genes associated with apoptosis were analyzed using reverse transcription polymerase chain reaction (RT-PCR), as illustrated in Figure 6a. Subsequently, Figure 6b illustrates that the concurrent administration of saccharin and caffeine led to a notably heightened up-regulation of the p53 and BAX genes in comparison with their individual treatments. While the individual treatments with saccharin or caffeine displayed a minimal impact on the expression of the Bcl-2 gene, the combined treatment demonstrated a modest down-regulation. However, the BAX/Bcl-2 ratio showed a significant increase.

## 3. Discussion

Among the major differences between normal cells and cancer cells is their ability to divide. Unlike normal cells, which control cell number homeostasis through the regulation of growth-promoting signals, cancer cells continuously divide by interfering with such signals [12]. Hence, inhibiting the abnormal division ability of these cancer cells may be an important point in cancer treatment [12]. As we can see in the results of our experiment, compared with the control group, the number of colonies generated in the single-treatment groups treated with saccharin or caffeine was reduced (*p* < 0.001). Furthermore, the number of colonies generated in the combination-treatment group treated with the combination of saccharin and caffeine was significantly reduced (*p* < 0.001) compared to the single-treatment groups. Based on this, it was confirmed that the combination of saccharin and caffeine inhibited the division of Ovcar-3 cells from single cells to colonies. It is considered that the saccharin–caffeine combination synergistically inhibited the proliferation and cell division of the ovarian cancer cells [13].

The evasion of apoptosis may contribute to tumor progression and to chemotherapy resistance [14]. Hence, the main mechanism of anticancer drugs is to suppress the formation of tumor cells by regulating the genes related to apoptosis in cancer cells [15]. Among these genes, p53, the Bcl-2 family, and caspase are known to be the factors regulating apoptosis in the endogenous pathway [16]. As a tumor suppressor gene, p53 is known to arrest cell cycle progression or induce apoptosis in response to external stress or stimuli such as DNA damage [17]. In this study, the combination treatment of saccharin and caffeine was associated with an increase in p53 expression, confirming that progress had been made in apoptosis.

Meanwhile, BAX is a pro-apoptotic factor belonging to the Bcl-2 family, and Bcl-2 is an anti-apoptotic factor [5]. BAX and Bcl-2 are balanced in a heterodimer form, and when the balance is disrupted, apoptosis is induced or inhibited [5]. As a result of our experiment, the expression of BAX was increased in the single-treatment groups compared to the control group, yet the expression was significantly increased in the combination-treatment group. When BAX is activated, the permeability of the mitochondrial membrane is increased, and cytochrome c is released into the cytoplasm [18]. The released cytochrome c forms an apoptosome with Apaf-1 to activate caspase-9, and the activated caspase-9 activates caspase-3 to induce apoptosis [19]. In this experiment, the expression of Bcl-2 was slightly down-regulated, but the ratio of BAX/bcl-2 was significantly increased, from which it can be concluded that cell death was promoted. However, the lack of protein-level analysis of the expression of apoptosis genes is a limitation of this study. 

Saccharin is part of the sulfonamide family, and various studies have already reported that such sulfonamides may be applied as a tumor treatment through inhibiting carbonic anhydrases [20]. Among the 15 carbonic anhydrase isoforms in the body, human carbonic anhydrases IX and XII are known to play a role in regulating the pH of cancer cells in the microenvironment formed around solid tumors [21]. It has been reported that saccharin inhibits the action of carbonic anhydrase IX, thereby inhibiting the growth of tumors due to the failure of pH control in the cancer cells [22,23,24,25].

Caffeine has been known to have antiproliferative effects on cancer cells through the activation of caspase-3 [14,15]. Caspase-3 is more frequently activated in an acidified environment, and when caspase is activated, the pH is known to decrease [26]. As saccharin inhibits carbonic anhydrase IX, the pH inside the cancer cells falls, which further increases the activity of caspase-3, and the cancer cells fail to regulate the pH, which induces apoptosis. It is important to note that the limitations of our study include the fact that we did not assess the activity of carbonic anhydrases and that we did not fully investigate the mechanism of synergism. Despite the limitations of this study, it may offer valuable insights into the potential synergistic effects of saccharin and caffeine in combination.

## 4. Materials and Methods

### 4.1. Cell Culture

The Ovcar-3 cell line used in the experiment was purchased from the American Type Culture Collection (ATCC, Manassas, VA, USA). Ten percent fetal bovine serum (FBS; Hyclone, Nelson, UK) and 1% penicillin–streptomycin were added to Roswell Park Memorial Institute-1640 (RPMI-1640; Hyclone, Nelson, UK) medium and cultured at 37 °C in 5% CO_2_, and the concentration was maintained.

### 4.2. Cell Viability Assay

We purchased the caffeine from Sigma-Aldrich Chemical (102177558, St. Louis, MO, USA). Saccharin was provided by JMC (JMC-Q-0340, JMC Corp., Seoul, Republic of Korea). The cell viability was measured using a water-soluble tetrazolium-8 assay (WST-8; BioMax, Seoul, Republic of Korea). Ovcar-3 cells were aliquoted in a 96-well plate at a concentration of 1 × 10^5^/mL cells and cultured for 24 h. After incubation for 24 h, the cells were treated with saccharin and caffeine for each concentration and reacted for 24 h. All experiments were conducted in RPMI-1640 medium at 37 °C and 5% CO_2_. After the reaction had proceeded for 24 h, 10 μL of WST-8 was added and reacted for 1–2 h. After the reaction, the absorbance was measured at 450 nm using a spectrophotometer (Thermo Fisher Scientific, Inc., Waltham, MA, USA).

### 4.3. Analysis of Cytotoxic Synergy

For confirmation of the synergistic effect, the Chou–Talalay method was used. Combination index (CI) values were calculated using CompuSyn software (ComboSyn, Inc., New York, NY, USA), with CI = 1 indicating an additive effect, CI < 1 indicating a synergistic effect, and CI > 1 indicating an antagonistic effect.

### 4.4. Clonogenic Assay

Ovcar-3 cells were cultured for 24 h at a concentration of 1 × 10^3^ cells/well in a 6-well plate. After incubation for 24 h, cells were treated with saccharin and caffeine alone and in combination for 24 h. After treatment, they were washed and incubated at 37 °C and 5% CO_2_ concentration for 14 d. Cells were washed with phosphate buffered saline (PBS) and fixed for 5 min at a methanol:acetic acid ratio of 3:1. Then, they were stained with 0.5% crystal violet dye for 15 min. The stained colonies were then washed with tap water, and the colonies were counted using ImageJ 1.49 software (U.S. National Institutes of Health, Bethesda, MD, USA).

### 4.5. Flow Cytometry Analysis of Apoptotic Cells Using Annexin V-FITC and Propidium Iodide (PI)-PE

The Ovcar-3 cells were cultured for 24 h at a concentration of 3 × 10^5^ cells/well in a 6-well plate. Cells were treated with saccharin and caffeine alone and in combination in the concentration range determined to be synergistic with the CI index value for 24 h. After washing with PBS, cells were collected via trypsin treatment and centrifuged. The supernatant was removed and treated with 1× binding buffer (Invitrogen, Carlsbad, MA, USA). Then, cells were treated with annexin V-FITC (Invitrogen) and pipetted, followed by reaction in the dark for 10 min. After reacting with annexin V-FITC for 10 min, they were washed with 1× binding buffer and, after centrifugation, only cells were left. Then, 1× binding buffer was added to react with PI-PE (20 μg/mL). Cells stained with annexin V-FITC and PI-PE were analyzed using a flow cytometer FACSCalibur™ (BD, Franklin Lakes, NJ, USA). The analysis of the results was performed using FlowJo 10.5.3 software (Tree Star Inc., Ashland, KY, USA).

### 4.6. Reverse Transcription Polymerase Chain Reaction (RT-PCR) Analysis

The RNA was extracted from the Ovcar-3 cells treated with saccharin and caffeine using GeneAll Ribospin (GeneAll, Valencia, CA, USA). The extracted RNA was quantified at 10 ngcompu, and one-step RT-PCR was performed using AccuPower^®^ Dual-HotStart™ RT-PCR PreMix (Bioneer, Daejeon, Republic of Korea). Each sample was amplified via targeting P53, BAX, Caspase-3, and GAPDH. Regarding the PCR conditions, cDNA was synthesized at 42 °C, and pre-denaturation was performed at 95 °C for 10 min. The PCR was conducted under the conditions specified in the corresponding reference studies [27,28,29]. The primers used for RT-PCR are presented in Table 1 [27,28,29]. The reactive product was electrophoresed on 2% agarose gel containing ethidium bromide (100 ng/mL). Product bands were photographed using the Cell Biosciences FluorChem E imager (Agilent, Santa Clara, CA, USA).

### 4.7. Statistical Analysis

Each experiment was independently replicated in triplicate. The results are expressed as the mean ± standard deviation (SD). Statistical analysis was conducted using one-way analysis of variance (ANOVA), followed by Dunnett’s multiple comparison test, utilizing GraphPad Prism 9.5.1 software (Boston, MA, USA). In all analyses, a significance level of *p* < 0.05 was considered statistically significant. Notably, significance levels were denoted as follows: *** *p* < 0.001, ** *p* < 0.01, and * *p* < 0.05, indicating comparisons with the corresponding control group.

## 5. Conclusions

In conclusion, the results of this study suggest that the combination of saccharin and caffeine may be more effective in suppressing the proliferation of Ovcar-3 cells and inducing apoptosis. This study may present the possibility of a new chemotherapeutic that can reduce drug toxicity and overcome anticancer drug resistance in the treatment of ovarian cancer patients.

## Figures and Tables

**Table 1:** The primer sequences used for RT-PCR.

Gene	Forward Primer Sequence, 5′-3′	Reverse Primer Sequence, 5′-3′	References
GAPDH	CATGACCACATCCATGCCATCACT	TGAGGTCCACCACCCTGTGCTGTA	[27]
P53	GAAGACCCAGGTCCAGATGA	CTCCGTCATGTGCTGTGACT	[27]
BAX	ACCAAGAAGCTGAGCGAGTGTC	ACAAAGATGGTCACGGTCTGCC	[28]
Bcl-2	TCGCCCTGTGGATGACTGA	CAGAGACAGCCAGGAGAAATCA	[29]

## Data Availability

The data are contained within the article.
